# The effect of remote fitting technology on hearing aid satisfaction

**DOI:** 10.1186/s43163-023-00447-7

**Published:** 2023-05-16

**Authors:** Bahtiyar Çelikgün, Furkan Büyükkal

**Affiliations:** 1 Oticon, Istanbul, Turkey; 2 Department of Audiology, School of Health Sciences, Istanbul Medipol University, Beykoz, 34810 Istanbul, Turkey

**Keywords:** Audiology, Hearing aid, Remote fitting, Smartphone tele-audiology, Telemedicine

## Abstract

**Background:**

Telemedicine is a method of providing remote healthcare services and consultations to patients using communication technology. Tele-audiology is a sub-branch of telemedicine. It refers to providing audiology services using telehealth strategies. This study aims to compare the satisfaction of patients who come to the hearing aid center and receive device fitting service and patients who have hearing aid fitting using tele-audiology service. For this purpose, hearing aid users were divided into two groups. The study group consisted of 17 participants (10 male, 7 females; mean age 65.17 ± 13.88) who continued fitting appointments remotely after the first clinic application, while the control group consisted of 23 participants (10 males, 13 females; mean age 62.17 ± 18.32) who had all hearing aid fittings performed face-to-face in the clinic. The participant’s satisfaction was assessed with The International Outcome Inventory for Hearing Aids Turkiye (IOI-HA-TR) questionnaire because it is practical and can be administered over the phone.

**Results:**

There were no significant differences in hearing aid satisfaction between those who came to the hearing center and filled out the IOI-HA-TR questionnaire personally and those who completed it through the Remote Care application (*p* < 0.05). In addition, most of the participants stated that using Remote Care solved their problems (35% very good, 24% good) and they were satisfied with the fitting of their hearing aids with this application (35% good, 29% very good). In addition, 13 out of 17 participants stated that they would pay attention to the “remote fitting” feature when purchasing a new hearing aid (76% very good). Moreover, they would like to continue the fitting using the Remote Care application (65% yes).

**Conclusion:**

Remote fitting technology via smartphone applications can facilitate the lives of hearing aid users and improve their quality of life in cases of risky conditions such as pandemics, various diseases, and physical limitations.

## Background

Telemedicine is a method of providing healthcare services and consultations to patients through the use of telecommunications technology, thus reducing personal contact for both the healthcare professional and the patient [1]. According to studies, the satisfaction of patients and health care professionals in tele-appointments is usually high [2–4].

Tele-audiology is a sub-branch of telemedicine. It refers to providing audiology services using telehealth strategies [5]. Nowadays, the use of tele-audiology has become more stable with advances in technology and telecommunication services. It has been successfully used in audiological screening, diagnostic tests, and hearing rehabilitation with hearing aids [6].

Tele-audiology was previously used for audiological assessment in rural areas [5]. It gained importance as a remote assessment tool with the onset of the pandemic. COVID-19 pandemic caused by the SARS-CoV-2 virus led to the declaration of a Public Health Emergency of International Concern in January 2020 and designation as a pandemic by the World Health Organization in March 2020 [7]. Due to the lack of knowledge on issues such as the contagiousness of this disease and its treatment, many institutions and organizations around the world have developed measures related to the isolation of people to reduce the spread of the disease [8]. In addition to the communication barrier created by hearing loss, isolation leads to a decrease in quality of life, related to reduced social activity and increased symptoms of depression [9].

Hearing aids are the most common solution to compensate for hearing problems, except for pathological conditions that can be treated surgically [10]. Wireless connections in hearing aids have also become indispensable tools for tele-audiology with the development of technology. Recently, audiologists have been able to remotely adjust their hearing aids via smartphones by connecting with users. In addition, with the onset of the pandemic, the use of tele-audiology for hearing aid fittings has increased to avoid some of the pandemic-related problems [11]. Moreover, remote-fitting may save time and money for both hearing aid users and the hearing care professionals [12].

At the present time, many hearing aid manufacturers offer remote, smartphone-based fitting protocols. However, while some users do not prefer to use smartphones, others are unable to use smartphones due to reasons such as vision problems, advanced age and/or inability to adapt to technology [6]. According to a survey of clinics that provide audiology services in the USA, they stated that they did not want to switch to tele-audiology at the beginning of the pandemic. However, after the extension of the pandemic was confirmed, another survey conducted revealed a significant increase in the use of tele-audiology [11].

As a result, tele-audiology provides convenience to both audiologists and patients. Nevertheless, the future of this technology depends on its adoption by users. It has been increasingly used throughout the world in recent years, but how much of it has been adopted in Turkiye is not known. Our study aimed to compare the satisfaction of patients who came to the hearing aid center for fitting with those who had their hearing aids adjusted using the tele-audiology service in Turkiye. We hope that our research will contribute to the literature on hearing aid users’ usage habits and their satisfaction with this technology.

## Methods

The study was performed with Oticon hearing aid patients using a Remote Care phone application in Turkiye. Hearing aid users were divided into two groups. Control group consisted of participants who had all hearing aid fittings performed face to face in the clinic, while study group consisted of continued fitting appointments remotely after the first clinic application. The inclusion criteria for the study group were as follows: use of Remote Care application at least once in the past 6 months, ≤ 80 years of age, slight to severe hearing loss between 26 and 90 dB HL, smartphone use, and a minimum of 1 year hearing aid experience. Similar inclusion criteria were accepted for control group as well except for Remote Care application use. The exclusion criteria were as follows for both groups: the presence of active ear disease, and cognitive or vision impairment that would preclude participation in the study. In addition, whereas the hearing aid functionality of the control group participants was checked at the hearing center, the study group was questioned on the phone to ensure that all participants are using their devices to fully functionality. A healthy phone conversation was essential for the study. Therefore, individuals with profound hearing loss were also not included in the study because they had lower discrimination scores and needed more visual cues than those with less hearing loss. The demographic information about the participants can be seen in Table 1.

**Table 1:** The demographic information about the participants

Groups	Age (mean)	Daily using time of the hearing aid					Type of fitting	
		*Never*	< *1 h*	*1–4 h*	*4–8 h*	*8* + *hours*	Bilateral	Unilateral
Study group	65.17 ± 13.88	0	0	0	2	15	16	1
Control group	62.17 ± 18.32	0	0	1	4	18	20	3

First, all of the hearing aid patient database was scanned at the Idea Hearing Systems Industry and Trade Inc (IDIS) hearing center in Turkiye, and 34 Oticon users who experienced remote fitting were identified. These users were called by an audiologist and two questionnaires were administered to those who wanted to participate in the study. Only 17 of the 34 participants agreed to participate (10 males, 7 females; mean age 65.17 ± 13.88). Seventeen users were not included in the study for different reasons. Seven hearing aid users did not agree to participate in the survey, one user died, two users were not available for phone conversation, three users could not be reached with the phone, and four users did not answer their phones. The International Outcome Inventory for Hearing Aids Turkiye (IOI-HA-TR) questionnaire was used as an assessment tool because it is practical and can be administered over the phone. In addition, in order to evaluate the experiences of the participants whose hearing aids were adjusted by remote care, a mini questionnaire consisting of 4 multiple-choice questions was applied to the participants over the phone (*see* Table 2).

**Table 2:** The sample of a short survey created for Remote Care application users

**Questions**						
Question 1	How well did the Remote Care program work in solving your problem?	Very Poor	Poor	Fair	Good	Very Good
Question 2	How satisfied are you with the setting made with the Remote Care program?	Very Poor	Poor	Fair	Good	Very Good
Question 3	How much do you pay attention to the Remote Care facility when buying a new hearing aid from now on?	Very Poor	Poor	Fair	Good	Very Good
Question 4	Do you want to continue the hearing aid adjustment with the Remote Care program from now on?	Yes	Sometimes	No		

Control group participants were randomly selected from hearing aid users who met the inclusion criteria in the IDIS clinics. The 23 users (10 males, 13 females; mean age 62.17 ± 18.32) who agreed to participate in the study were given an information form, and their consent was obtained. They were then asked to complete the IOI-HA-TR questionnaire.

In IDIS hearing aid clinics, the first appointment of all hearing aid users is performed face-to-face. The first fitting is completed with the Real Ear Measurement (REM) verification method according to the NAL-NL 2 prescription formula for all patients. In addition, IDIS clinics have a standard control procedure. If users do not want an additional appointment, routine controls are performed in the 1st month, 3rd month, 6th month, and 1st year. Participants of both groups were followed online or face-to-face similarly in terms of hearing aid satisfaction.

The Statistical Package for the Social Sciences program was used for the analysis. Since the data were not distributed normally, non-parametric test methods were used. The Mann–Whitney *U* test was used to compare the two groups. In addition, Pearson correlation was used to correlate the questions where necessary.

This study was approved by the Ethics Committee of Istanbul Medipol University (registration no. 7834E184XA).

## Results

Thirty-seven hearing aid users where experts at IDIS made adjustments remotely using Remote Care were contacted, and seventeen of them were provided to answer the surveys. In addition, 23 hearing aid users who have never used Remote Care were included in the study as the control group. When the participants in the two groups were compared in terms of their mean age, no significant difference was found (*p* < 0.05).

There was no significant difference in the answers to any questions for those who came to the hearing aid center and completed the IOI-HA-TR questionnaire in person or those who completed it with Remote Care (*p* < 0.05) (*see* Table 3).

**Table 3:** Comparison of IOI-HA-TR questionnaire with Mann–Whitney *U* test

Comparison of IOI-HA-EN Questionnaire with Mann–Whitney ***U*** test			
**Questions**	Groups	Mean ± SD	*P*
Question 1	Study group	4.73 ± 0.54	0,808
	Control group	4.82 ± 0.39	
Question 2	Study group	4.17 ± 0.77	0,787
	Control group	4.23 ± 0.83	
Question 3	Study group	3.73 ± 0.75	0,120
	Control group	3.29 ± 1.10	
Question 4	Study group	4.00 ± 0.85	0,850
	Control group	3.94 ± 1.24	
Question 5	Study group	4.39 ± 0.83	0,401
	Control group	4.70 ± 0.46	
Question 6	Study group	4.39 ± 0.72	0,725
	Control group	4.29 ± 1.15	
Question 7	Study group	4.30 ± 0.70	0,935
	Control group	4.23 ± 0.97	

*SD* standard deviation

Table 4 shows a negative correlation between the IOI-HA-TR questionnaire results in questions 1 and 4 of old and young participants. According to the first question, older participants in the study group used hearing aids for a shorter period than younger participants (*Think about how much you used your present hearing aids over the past two weeks*). *On an average day, how many hours did you use the hearing aids?*) (*r* (15) =  − 0.436, *P* = 0.038). However, there was no significant difference in control group (*p* = 0.395). In question 4, the hearing aid satisfaction of older participants in the study group was lower than that of younger participants (*Considering everything, do you think your present hearing aids are worth the trouble*?) (*r* (15) =  − 0.421, *P* = 0.046).

**Table 4:** Comparison of IOI-HA-TR questionnaire with correlation test

Correlation of IOI-HA-TR survey questions with age			
*Questions*	Groups	*P*	*r*
Question 1	Study group	0.038^a^	− 0.436
	Control group	0.395	− 0.221
Question 4	Study group	0.046^a^	− 0.421
	Control group	0.081	− 0.435

^a^Significant differences

The average duration of using the last purchased hearing aids of the participants in the study group was 29.9 months while in the control group was 24 months. The long-term users of current hearing aids in the study group were using their hearing aids less during the day than those who purchased their hearing aids recently (*r* (15) =  − 0.509, *P* = 0.013). In the control group, the short-term users of current hearing aids were using them for longer during the day (*r* (15) = 0.579, *P* = 0.015) (*see* Table 5). In addition, according to question 7, participants in the study group stated that as they spent more time with their current hearing aids, their enjoyment of life decreased. (*Considering everything, how much has your present hearing aids changed your enjoyment of life?*) (*r* (15) =  − 0.442, *P* = 0.035).

**Table 5:** Correlation of IOI-HA-TR survey questions with current hearing aid use time

Correlation of IOI-HA-TR survey questions with current hearing aid use time			
*Questions*	Groups	*P*	*r*
Question 1	Study group	0.013^a^	− 0.509
	Control group	0.015^a^	0.579
Question 7	Study group	0.035^a^	− 0.442
	Control group	0.420	0.290

^a^Significant differences

**Fig. 1:**
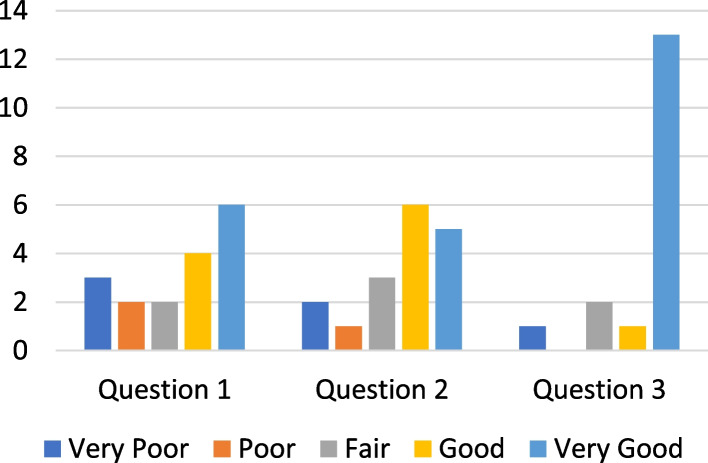
The results of the short survey created for Remote Care application users

**Fig. 2:**
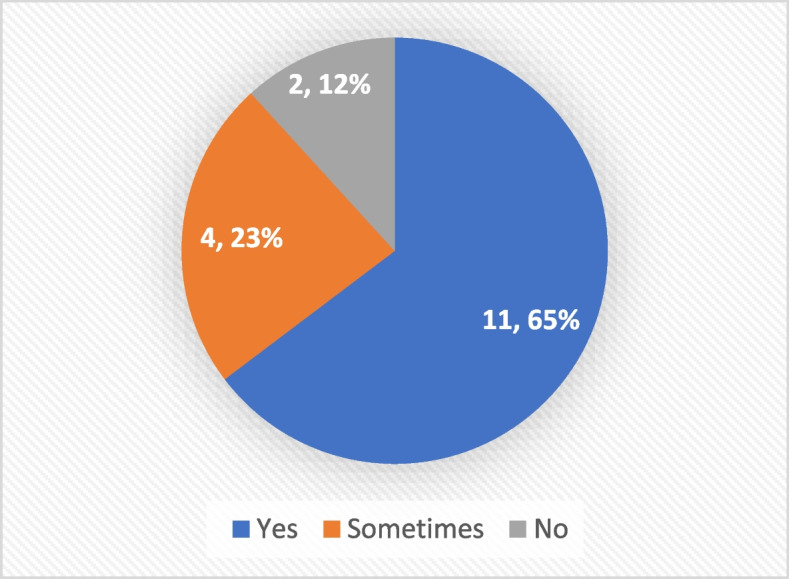
The distribution of answers to question 4

We have developed a short survey to get the opinions of those who used remote fitting. The survey results are shown in Figs. 1 and 2. When the questions are examined, the survey answers of the users who used Remote Care were mostly positive (*see* Fig. 1). Most of the participants stated that using Remote Care solved their problems in question 1 (35% Very good, 24% good), and they were satisfied with the fitting of their hearing aids with this application according to question 2 (35% good, 29% very good). In addition, 13 out of 17 participants stated that they would pay attention to the “remote fitting” feature when purchasing a new hearing aid in question 3 (76% very good). In question 4, when the participants were asked whether they would like to continue the fitting using the Remote Care application, 65% of the participants answered “yes” (*see* Fig. 2).

## Discussion

Telemedicine allows patients to find solutions to their problems on an online platform without leaving their homes or office. According to some studies in the literature, telemedicine technology can be effectively used in audiology, especially for hearing aid fitting. In a study conducted by Wesendahl in 2003, it was stated that the hearing aid fitting can be made remotely and this will save time and money [12]. In addition, teleconsultation was found an efficient procedure for hearing aid programming, verification, and fitting when face-to-face services were not available [13]. The use of mobile applications in health has increased with the development of smartphone technology. Sarkar et al. (2016) showed that mobile applications can be very useful in various chronic diseases such as diabetes and depression [14]. Mobile applications are also used in audiology. In a study conducted in 2022, patients’ hearing loss was examined remotely by using a mobile application instead of a sound booth. Although the hearing threshold results were not as correct as those of the test performed in a quiet cabin, there was a significant correlation [15]. Also, audiologists were willing to use a smartphone application for the assessment of patients [16].

In recent years, hearing aid manufacturers have also developed various applications, offering remote hearing aid fitting opportunities for users. The Remote Care application has been developed by Oticon and offers remote settings for users (Oticon WP). Since the app can work with all Oticon hearing aids compatible with 2.4 GHz, we were able to include hearing aids from different segments and models in our study. However, despite the technological development and the negative impact of the COVID-19 pandemic, the use of Remote Care in Turkiye was limited. The database of the IDIS Hearing Center, which has 35 branches in Turkiye, was searched and only 34 users who used Remote Care actively were found. Seventeen of them could be reached by phone and agreed to participate in the study. Due to the small number of Remote Care users in Turkiye, we had to perform our study with a limited study group. In the future, research with larger participants will contribute to the literature.

Participants’ hearing aid satisfaction was evaluated with the IOI-HA-TR questionnaire [17], and no difference was observed between remote and face-to-face fitting. This result is consistent with Convery et al. [18]. They compared two groups to understand the effect of tele-audiological hearing aid fitting on users; fitting on app and face-to-face fitting. They used to Satisfaction with Amplification in Daily Life scale (Cox and Alexander, 1999) for assessment of participants’ hearing aid satisfaction [19]. In finally, no significant difference was observed between the two groups. In our study, we also used a short 4-question survey to understand users’ attitudes towards remote fitting technology, and we found that users were largely willing to use this technology. Convery et al. used a similar short survey, and they concluded that care through the smartphone app has no harmful effects, at least in the short term, and may allow patients to communicate remotely with their audiologist to receive help with hearing aid problems [18]. Also, It was performed a study measuring hearing aid satisfaction with the IOI-HA questionnaire among participants who remote and office-based hearing aid renewal. Surprisingly, the remote fitting group had lower satisfaction scores than the face-to-face group. However, the authors compared the results of the remote fitting group with data from a Swedish nationwide database and stated that the results were not statistically significant. As a result, they emphasized that remote fitting is also useful in hearing aid renewal [6].

In addition to the online fitting service provided by an audiologist, there are also self-fitting options tried and offered by different manufacturers. In a recent study, it was stated that the digital self-fitting tool enables multiple sessions and easy re-fitting, with the potential to outperform the classical fitting approach. In the following years, in addition to the online fitting method, the self-fitting method may often take place in tele-audiology [20].

Remote Care can install easily on the smartphone and acts as a bridge between the hearing aid and hearing care professional. Despite the easing of the pandemic conditions, this technology continues with 44 active hearing care specialist users in our country. During to study, it was observed that some users who participated this study purchased new smartphones in order to use Remote Care application. On the other hand, this technology also requires a smartphone and a stable Internet connection to work. However, some users, especially elderly users, may not want to use a smartphone. In addition, the use of smartphones can also be restricted by physical disabilities, such as hand tremors and vision difficulties. In our study, no significant difference was found between the Remote Care fitting group and the mean age of the face-to-face fitting group. However, in the remote care group, it was found that older users were less satisfied with their hearing aids and tended to use them less. This may be due to the mental and physical limitations of aging [21].

The long-term users of current hearing aids in Remote Care group were using their hearing aids less during the day than those who purchased their hearing aids recently. This may be due to the fact that individuals with hearing loss express themselves more limitedly on the online platform during the fitting appointment. Obviously, it is known that even individuals with unilateral hearing loss are adversely affected by their expressive language skills [22]. In addition, online hearing aid fine-tuning can also be difficult for hearing care professionals because they cannot fully see the user’s body language. It is well known that non-verbal communication affects the effectiveness of the verbal communication [23]. Moreover, enjoyment of life may decrease as participants spent more time with their insufficient tuned current hearing aids.

In our study, we used a short survey to assess the impact of remote fitting technology on users. Because there is no structured, valid-reliable questionnaire on this subject in the literature. A “comprehensive” questionnaire on tele-audiology would be beneficial for future studies. In addition, repeating our research with a larger experimental group will provide more reliable results. Moreover, the longitudinal study design with certain periods can also give information about the adaptation process of Remote Care users. Furthermore, the comparison of the groups with objective measures such as Real Ear Measurement will enable the evaluation of more different variables.

## Conclusions

There is no significant difference between patients who fitted hearing aids face-to-face and those who received them remotely. Patients can express themselves more clearly when they are face-to-face with an audiologist and can be examined in greater detail. However, it is also possible that hearing aid users could not go to the hearing center due to the risk of COVID-19, rules related to the pandemic or physical limitations. Nonetheless, without remote fitting protocol, these patients may had been completely deprived of functional hearing aids. The possibility of remote hearing aid adjustment with a smartphone application can improve the life quality of hearing aid users in cases of pandemics, various diseases, and physical limitations.

## Data Availability

The data analyzed and created during the study are not included in the submission; all data are reserved by the corresponding author. However, when necessary, the corresponding author will share the data with the journal.

## Abbreviations

IDIS: Idea Hearing Systems Industry and Trade Inc.
IOI-HA-TR: The International Outcome Inventory for Hearing Aids Turkiye
